# Lignocellulose adaptation drives polysaccharide biosynthesis in *Tremella fuciformis*: metabolomic and proteomic insights into CAZyme regulation

**DOI:** 10.3389/ffunb.2025.1617458

**Published:** 2025-07-11

**Authors:** Yingyin Xu, Qian Dong, Shilin Zhang, Liyuan Xie, Qian Zhang, Xueqin Shu, Jie Zhou, Weihong Peng

**Affiliations:** ^1^ National-Local Joint Engineering Laboratory of Breeding and Cultivation of Edible and Medicinal Fungi, Sichuan Institute of Edible Fungi, Sichuan Academy of Agricultural Sciences, Chengdu, Sichuan, China; ^2^ Scientific Observing and Experimental Station of Agro-microbial Resource and Utilization in Southwest China, Ministry of Agriculture, Chengdu, Sichuan, China

**Keywords:** *Tremella fuciformis*, lignocellulose in growth substrates, polysaccharide content, CAZyme, metabolomic and proteomic insights

## Abstract

**Background/Objectives:**

*Tremella fuciformis* is an edible fungus prized for its culinary value. The polysaccharide content of *T. fuciformis* grown on a Cyclobalanopsis substrate (TY3) was significantly higher than those grown on a mixed substrate (TF1) made of wheat bran and cottonseed hull.

**Methods:**

Metabolomics and proteomics were used to assess the effects of lignocellulose (consisting of cellulose, hemicellulose, and lignin) in different growth substrates on the polysaccharide content of *T. fuciformis* and its formation mechanism.

**Results:**

TY3 had a higher lignocellulose content than TF1. The metabolites of carbohydrates and carbohydrate conjugates in TY3-grown specimens were significantly upregulated. Among the 21 identified metabolic pathways with enriched proteins, carbohydrate metabolism was the most enriched. The Carbohydrate-Active Enzyme (CAZyme) database was used to annotate 161 carbohydrate enzymes, and 67 of them were differentially expressed proteins. Carbohydrate synthetases were upregulated much more using TY3.

**Conclusions:**

*Tremella fuciformis* grown on TY3 was verified to possess a lower ability for lignocellulose degradation (as evidenced by decreased synthesis of cellulase, xylanase, and lignin peroxidase) but a stronger ability for carbohydrate synthesis (as evidenced by increased synthesis of cellulose and hemicellulose). Our study enhances the control of polysaccharide content in *T. fuciformis*, thereby facilitating its processing for food applications.

## 1 Introduction

Tremella fuciformis, which is also known as white or silver fungus, is an edible mushroom used to make tremella soup (which is widely consumed in traditional Chinese cuisine). Tremella fuciformis can also be used as a food additive for its medicinal effects, improving immunodeficiency (Shi et al., 2014), modulating the gut microbiome (Xu et al., 2021), and acting as antitumor (Chen, 2010) and anti-aging agents (Shen et al., 2017).

Tremella fuciformis is currently grown in two ways, each resulting in mushrooms with different characteristics. Tremella fuciformis produced in the traditional way has a higher polysaccharide content and viscosity of the subsequently produced soup, compared to industrially produced T. fuciformis. The industrial cultivation method involves growing mushrooms on a substrate composed mainly of wheat bran and cottonseed hulls, with T. fuciformis harvested bimonthly. This cultivation method accounts for more than 90% of all T. fuciformis production. The other method used is the traditional cultivation approach. In this case, the fungi were allowed to develop naturally on Cyclobalanopsis bed logs and were harvested once a year (Huang et al., 2021). However, consumers prefer the produced soup of T. fuciformis to be thick, which is closely associated with the higher polysaccharide content of the T. fuciformis fruit body (Jing et al., 2019). Due to the long cycle, high cultivation cost, and low yield, the supply available for the traditional cultivation approach is very small. The inability to provide T. fuciformis with a high polysaccharide content, thick soup produced, and high yield has become a bottleneck problem restricting the high-quality development of the T. fuciformis industry.

To address this issue, the same variety of T. fuciformis grown using a mixed substrate TF1 (made of wheat bran and cottonseed hull) and a traditional substrate TY3 (Cyclobalanopsis log) was collected. Many researchers have declared that different growth substrates affect the quality of the final product (Culleton et al., 2013; Saritha et al., 2016; Keller, 2019). We hypothesize that different substrates (containing different contents of cellulose, hemicellulose, lignin, and pectin) lead to the production of different enzymes to catalyze the metabolic pathways in T. fuciformis and thus affect the polysaccharide content and viscosity of the soup produced therefrom. Integrated proteomics and metabolomics analysis was used to identify the enzymes involved in the metabolism of carbohydrates and the function of enzymes annotated with CAZyme databases. By understanding the properties governing the polysaccharide content and formation mechanism using these two types of growth substrates, a theoretical basis was provided for the development of a high polysaccharide content and efficient cultivation technology system for T. fuciformis. Exploration of functional genes or proteins that affect the polysaccharide content and the increase of the polysaccharide content in the fruiting body can offer an important target for the molecular breeding of excellent strains.

## 2 Materials and methods

### 2.1 Preparation of substrates and *Tremella fuciformis*

Samples of mixed substrate (30% wheat bran and 70% cottonseed hull) and *Cyclobalanopsis* log substrate were prepared, and the mixed substrate was put into bottles. The same culture of *T. fuciformis* and *Annulohypoxylon stygium* was inoculated into bottles and logs, respectively. Both samples developed at 25°C until the fruit bodies were harvested. *Tremella fuciformis* SMCC174.01.1 and *A. stygium* SMCC238.02.34 were isolated and preserved in our lab.

### 2.2 Determination of components and enzyme activity

The polysaccharide content of the fungi was determined using a colorimetric method using phenol and sulfuric acid (Dubois et al., 1956). The cellulose, hemicellulose, pectin, and lignin contents of the growth substrates were tested using commercial kits (BC4280, BC4440, BC1400, and BC4200; Beijing Solarbio Science & Technology Co., Ltd.). ELISA tests were also performed using commercial kits (Shanghai Huabang Biotechnology Co., Ltd.) to measure glycosyltransferase activity. The activity of pectin methylesterase (PME) was measured using commercial kits from Shanghai C-reagent Biotechnology Co., Ltd. under the conditions of 37°C and pH 7.8. Xylanase activity was identified using commercial kits from Jiangxi Jiangnanchun Biotechnology Co., Ltd. The samples were incubated at 40°C for 60 min with reagents 1 and 2 and then in a boiling water bath for 5 min. After cooling, distilled water was added to measure the OD value at 540 nm, and finally, xylanase enzyme activity was calculated. The activity of cellulase (endoglucanase) and lignin peroxidase was also investigated using commercial kits (BC2540 and BC1610; Beijing Solarbio Science & Technology Co., Ltd.). The sample was mixed with reagents 1 and 2 evenly, then bathe in a 40°C water bath for 30 min. After taking it out, it was immediately put into boiling water and boiled for 15 min. Then, reagent 3 was added and boiled in boiling water to develop color. After cooling, distilled water was added to measure the absorbance value at 540 nm, and finally, cellulase activity was calculated. Reagents 1, 2, and 3 were mixed in a ratio of 6:2:1 to form a working solution, which was preheated at 37°C. After adding the sample and the working solution, the OD values were measured at 310 nm at different reaction times to calculate lignin peroxidase activity.

### 2.3 Characteristics of *Tremella fuciformis* soup

Fresh samples of T. fuciformis fruit bodies (30 g) were added to water (2 L) and boiled for 30 min. The T. fuciformis soup was then filtered (using a 40-mesh sieve). The supernatant was used to fill two-thirds of a test cup for texture analysis; the weight of the filtered colloids was also recorded. A texture analyzer was used in compression mode to determine the texture of the T. fuciformis soup. The experimental parameters used were 35-mm diameter A/BE probe, 10 kg load cell capacity, returning to start option, testing speed of 1 mm/s, post-test speed of 2 mm/s, target distance of 25 mm, auto (force) 5 g of trigger type, and data acquisition rate of 250 pps. The firmness, consistency, cohesiveness, and work of cohesion of this soup were tested.

Firmness: This refers to the force corresponding to the largest positive peak on the curve. Firmness indicates the hardness of the surface of the T. fuciformis soup. Consistency: This pertains to the average of the positive peak area of the curve and the yield point to the maximum value. Consistency refers to the internal friction resistance to fluid deformation, reflecting the fluidity of the T. fuciformis soup. The greater the consistency, the worse the fluidity. Cohesion: This refers to the force value corresponding to the largest negative peak on the curve. Cohesion pertains to the size of the internal binding force required to maintain the shape of the sample, reflecting the strength of the intermolecular binding effect of the T. fuciformis soup. The greater the value, the larger the cohesion. Viscosity: This refers to the negative peak area of the curve. Viscosity is related to flow resistance, which reflects the resistance of the T. fuciformis soup when it flows. The larger the value, the greater the viscosity.

### 2.4 Metabolomics analysis

Tremella fuciformis fruit bodies (100 mg) were ground using liquid nitrogen. The homogenate was then resuspended with prechilled 80% methanol and centrifuged at 15,000*g*. The supernatant was diluted with mass spectrometry water until the methanol content was 53%. Then, it was centrifuged at 5,000 rpm and 4°C for 1 min, and the supernatant was transferred into a new centrifuge tube and freeze-dried into a dry powder. A 10% methanol solution was added according to the sampled volume to dissolve the powder and then injected into a UPLC-MS/MS system for analysis.

UPLC-MS/MS analysis was performed at the Novogene facility in Beijing using a Vanquish UHPLC system (Thermo Fisher, Germany) coupled with an Orbitrap Q Exactive™ HF mass spectrometer (Thermo Fisher, Germany). The samples were injected into a Hypersil GOLD column (100 mm × 2.1 mm, 1.9 μm) with a 17-min linear gradient and a flow rate of 0.2 mL/min. In a positive polarity mode, a 0.1% formic acid (FA) solution (eluent A) was used followed by methanol (eluent B); in a negative polarity mode, eluent A (5 mM of ammonium acetate, pH 9.0) was used first followed by eluent B (methanol). The solvent gradients employed were as follows: 2% B for 1.5 min, 2%–85% B for 3 min, 100% B for 10 min, 100%–2% B for 10.1 min, and 2% B for 12 min, successively. The Q Exactive™ HF mass spectrometer was used in a positive/negative polarity mode with a capillary temperature of 320°C, spray voltage of 3.5 kV, sheath gas flow rate of 35 arb, auxiliary gas flow rate of 10 arb, S-lens RF level of 60, and auxiliary gas heater temperature of 350°C. The raw UHPLC-MS/MS data were analyzed using Compound Discoverer 3.1 software (CD3.1, Thermo Fisher) to conduct peak alignment, peak picking, and quantitation of each metabolite. The software packages R (v3.4.3), Python (v2.7.6), and CentOS (v6.6) were used to conduct the statistical analyses. The metabolites were annotated using three databases: KEGG (https://www.genome.jp/kegg/pathway.html), HMDB (https://hmdb.ca/metabolites), and LIPID MAPS (http://www.lipidmaps.org/). Partial least-squares discriminant analysis (PLS-DA) was performed using metaX (Wen et al., 2017). Univariate analysis (*t*-test) was used to calculate statistical significance (*p*-value). Metabolites with variable importance in projection (VIP) values >1, *p*-value <0.05, and fold change (FC) ≥2 or ≤0.5 were deemed to be differentially abundant metabolites (DAMs). The data used to generate clustered heatmaps were first normalized using the *z*-scores of the intense areas of the differential metabolites. The data were then plotted using the heatmap function in the R package. A *p*-value <0.05 was considered to be statistically significant, and correlation plots were plotted using the corrplot package in R. The KEGG database was used to identify the functions of these metabolites and their metabolic pathways. Metabolic pathways satisfying the condition *x*/*n* > *y*/*N* were considered to be enriched (and those with a *p*-value <0.05 were also deemed to have been significantly enriched).

### 2.5 Proteomics analysis

Additional samples were individually ground in liquid nitrogen lysed with SDT lysis buffer and then subjected to ultrasonication on ice for complete dissolution. The samples were then incubated at 95°C for 8 min and then centrifuged at 12,000*g* for 15 min at 4°C. The resulting supernatant was collected, 10 mM of DTT was added, and then incubated at 56°C for 1 h. Sufficient IAM was added and incubated at room temperature in the dark for 1 h. Next, four volumes of precooled (–20°C) acetone were added and precipitated at −20°C for at least 2 h. The samples were centrifuged at 4°C and 12,000*g* for 15 min, and the precipitate was collected. One milliliter of precooled (−20°C) acetone was added during the resuspension and washing of the precipitate. The mixture was centrifuged at 12,000*g* for 15 min at 4°C. The precipitate was collected and air-dried, and the protein precipitate was dissolved using an appropriate amount of protein dissolution solution (8 M of urea, 100 mM of TEAB, pH = 8.5). BSA protein solution was used as a standard in the protein concentration tests (performed using 12% SDS-PAGE gel electrophoresis). Qualified protein samples were dissolved in DB. After digestion using trypsin and CaCl_2_, the supernatant was loaded onto a C18 desalting column and washed with buffer eluent.

More precisely, a 0.1-M solution of tetraethylammonium bromide (TEAB) buffer was supplemented to the samples, along with a tandem mass tag (TMT) labeling reagent dissolved in acetonitrile. All the labeled samples were mixed in equal volumes, desalted, and lyophilized. For separation, a gradient elution was established utilizing mobile phases: phase A comprised 2% acetonitrile, adjusted to pH 10.0 with ammonium hydroxide, and phase B consisted of 98% acetonitrile. Fractionation of the samples was conducted on a Rigol L3000 HPLC system equipped with a C18 column (Waters BEH C18, dimensions 4.6 mm × 250 mm, particle size 5 μm), with the column oven maintained at 45°C. All resultant fractions were dried under vacuum and reconstituted in 0.1% (v/v) aqueous formic acid (FA). These fractions were then subjected to shotgun proteomics analysis using an EASY-nLC 1200 UHPLC system (Thermo Fisher Scientific), interfaced with a Q Exactive HF-X mass spectrometer (Thermo Fisher Scientific) operated in the data-dependent acquisition (DDA) mode.

The samples were injected into a homemade C18 Nano-Trap column (dimensions: 4.5 cm × 75 μm, 3 μm). The subsequent peptide separation was achieved in a homemade analytical column (15 cm × 150 μm, 1.9 μm), using a linear elution gradient. The separated peptides were then analyzed using a Q Exactive™ HF-X mass spectrometer (Thermo Fisher) fitted with a Nanospray Flex™ ion source. The spray voltage was adjusted to 2.3 kV, while the ion transfer capillary temperature was maintained at 320°C. A full scan encompassed the *m*/*z* range from *m*/*z* 350 to 1,500 with a resolution of 60,000 (at *m*/*z* 200). The target value of the automatic gain control (AGC) was set to 3 × 10^6^, and the maximum ion injection time was 20 ms. Within the full scan, the top 40 most abundant precursors were selected for fragmentation via higher-energy collisional dissociation (HCD) and subsequent MS/MS analysis, where the resolution was 45,000 (at *m*/*z* 200) for 10 plex. The AGC target was set to 5 × 10^4^, with a maximum ion injection time of 86 ms; the normalized collision energy was set to 32%; the intensity threshold was set to 1.2 × 10^5^; and the dynamic exclusion parameter was configured to 20 s.

Proteome Discoverer (PD) software (v2.4, Thermo Fisher) was used to analyze the spectra obtained in each run and compare the results with the UniProt database. The mass tolerance for the precursor ion was set to 10 ppm, and that for the product ion to 0.02 Da. Carbamidomethyl was specified as a fixed modification, and oxidation of methionine (M) and TMT plex were specified as dynamic modifications. Acetylation, TMT plex, Met-loss, and Met-loss+Acetyl were specified as N-terminal modifications in PD. A maximum of two missed cleavage sites was allowed. The software PD was used to further filter the retrieval results to improve the quality of the analysis results. The protein quantitation results were statistically analyzed using *t*-tests. Proteins whose quantitation results were significantly different in the experimental and control groups (*p* < 0.05 and FC > 1.2 or FC < 0.83) were assumed to be differentially expressed proteins (DEPs). The InterProScan program against the non-redundant protein database was used for Gene Ontology (GO) functional analysis (Jones et al., 2014). The KEGG and Clusters of Orthologous Groups (COG) databases were used to study the protein families and pathways. Carbohydrate-active enzyme was annotated using the dbCAN2 meta server (Zhang et al., 2018). Raw omics data have been uploaded to iProX (IPX0012156000) and MetaboLights (REQ20250604210974).

### 2.6 Statistical analysis

Data met the assumptions of normality (Shapiro–Wilk *p* > 0.05) and homogeneity of variances (Levene’s *p* = 0.18). Differences among groups were analyzed by one-way ANOVA with Tukey’s *post-hoc* test. To account for multiple comparisons across three experimental conditions, *p*-values were adjusted using the Benjamini–Hochberg procedure (FDR = 0.05).

According to the calculation formula (*x*/*n* > *y*/*N*), the 21 most enriched metabolic pathways were selected. *x* represents the number of DEPs associated with this pathway, *y* represents the number of all proteins associated with this pathway, *n* represents the number of DEPs annotated by KEGG, and *N* denotes the number of all proteins annotated by KEGG.

The Pathview package was used to co-analyze the proteome–metabolome data. After supplying Pathview with gene or compound data and specifying the target pathway, the software automatically downloaded the pathway graph data, parsed the data file, mapped the user data to the pathway, and rendered a pathway graph containing the mapped data (Luo and Brouwer, 2013). The software packages R (v3.4.3), ggplot2 (v3.3.4), and grid (v3.4.3) were used to harvest bubble diagrams of the different proteins and metabolites both enriched in the KEGG pathways. The software packages R (v4.0.3), ggplot2 (v3.3.5), and pathview (v1.30.1) were used to identify the different enzymes and metabolisms involved in the same pathways.

All the results presented are expressed in the form: means ± standard deviation (SD). Differences between groups are deemed to be statistically significant if *p <*0.05.

## 3 Results

### 3.1 Characteristics of TF1 and TY3 and *Tremella fuciformis* grown on them

#### 3.1.1 Cellulose, hemicellulose, pectin, and lignin contents of the substrates

**Figure 1:**
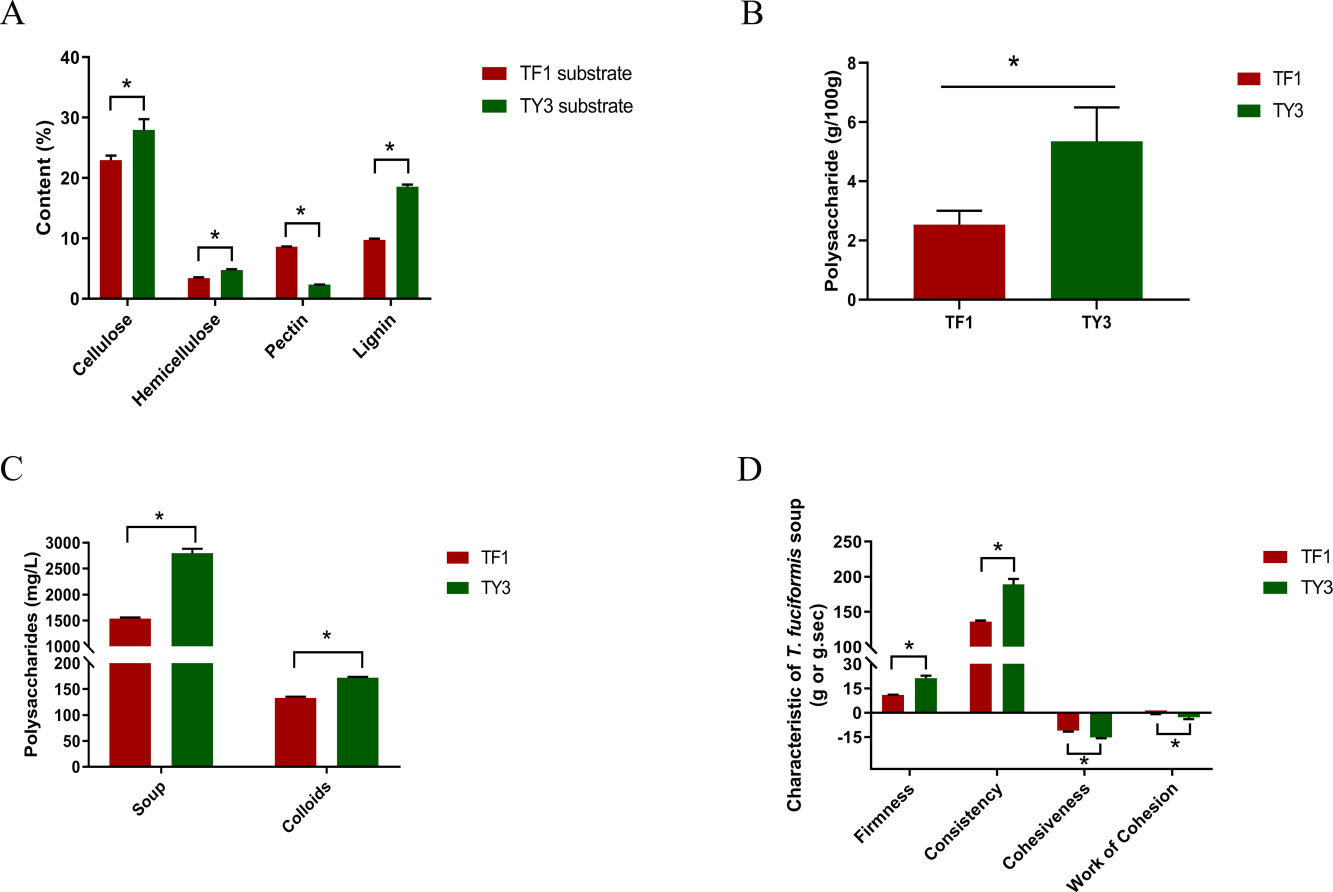
Characteristics of the substrates and *Tremella fuciformis* grown on them. **(A)** Cellulose, hemicellulose, pectin, and lignin contents of the substrates. **(B)** Polysaccharide content of *T. fuciformis* grown on TF1 and TY3. **(C)** Polysaccharide contents of the *T. fuciformis* soups and their colloids. **(D)** Textural characteristics of the *T. fuciformis* soup produced. The numerical values correspond to mean  ±  SD (three replications), and significant differences (i.e., *p* < 0.05) between the TF1 and TY3 results are marked using asterisks.

The carbohydrate compositions of different growth substrates naturally exhibit variations. Specifically, the cellulose, hemicellulose, pectin, and lignin contents of substrates TF1 and TY3 differed significantly (**Figure 1A**). In both substrates, cellulose was the predominant component, followed by lignin. Notably, *Cyclobalanopsis* substrate TY3 contained higher levels of cellulose, hemicellulose, and lignin but lower levels of pectin compared to the mixed substrate TF1. The pectin content in TF1 was 3.7 times that of TY3, while its lignin content was only 52.4% of TY3’s.

#### 3.1.2 Polysaccharide content of *Tremella fuciformis* and the soup produced

**Figure 1B** illustrates the polysaccharide content in the fruiting bodies of *T. fuciformis* grown on different substrates. Additionally, the polysaccharide content in the liquid and colloid phases of the soup was measured, with the results presented in **Figure 1C**. Textural analysis results, shown in **Figure 1D**, characterize the viscosity of the *T. fuciformis* soup. As indicated in **Figure 1**, the polysaccharide content of *T. fuciformis* grown on the mixed substrate TF1 is only 47.3% of that observed in specimens grown on *Cyclobalanopsis* substrate TY3. Similarly, the polysaccharide content in both the liquid and colloid phases of the soup produced from TF1-grown *T. fuciformis* was significantly lower than that of the soup made from TY3-grown fungi. Moreover, the firmness, consistency, cohesiveness, and stickiness of the soup produced from TF1-grown *T. fuciformis* were significantly lower than those of the soup made from TY3-grown specimens. This suggests that the soup derived from TY3-grown fungi demonstrates superior rheological properties. Generally, higher sugar content in a solution correlates with increased viscosity (Sun et al., 2018), indicating that *T. fuciformis* grown on substrate TY3 has a higher polysaccharide content and produces a more consistent soup.

### 3.2 Overview of the metabolomics results

To investigate the differences between the *T. fuciformis* cultivated in the two different substrates, the metabolite profiles of TF1- and TY3-grown specimens were analyzed and quantitatively compared using LC-MS/MS (in both positive and negative ionization modes). As a result, 1,221 metabolites were identified.

**Figure 2:**
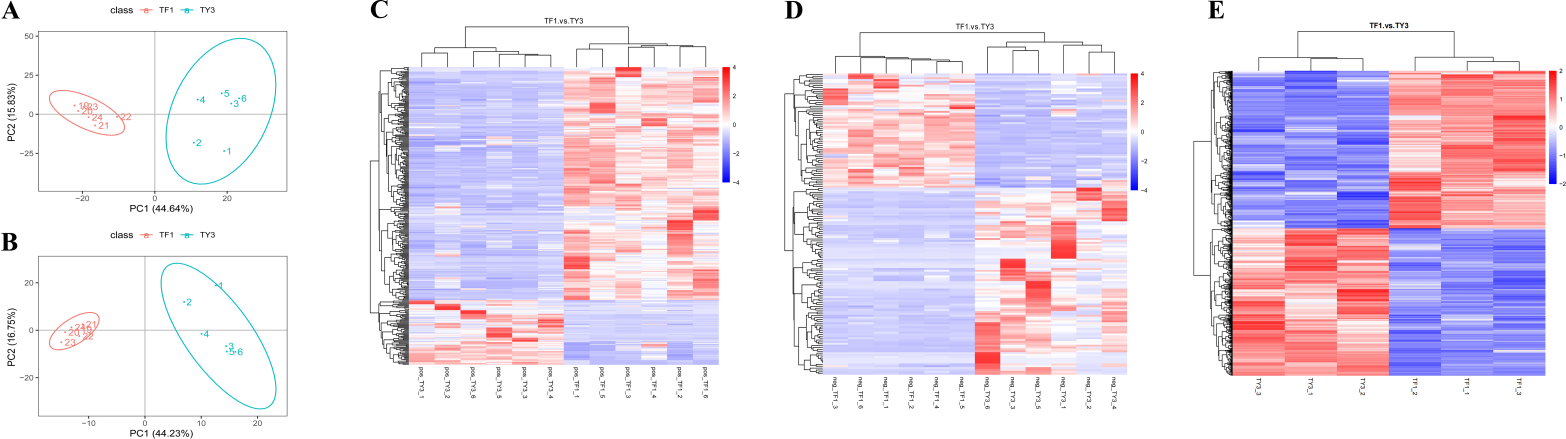
Analysis of the DAMs and DEPs in *Tremella fuciformis*. **(A)** PCA results for the DAMs in the positive ionization mode. **(B)** PCA results for the DAMs in the negative ionization mode. **(C)** Hierarchical clustering heatmap of the DAMs in the positive ionization mode. **(D)** Hierarchical clustering heatmap of the DAMs in the negative ionization mode. **(E)** Clustering heatmap of the DEPs.

The PLS-DA results (**Figures 2A, B**) show that the metabolic profiles of TF1- and TY3-grown specimens were significantly different in general. In total, 488 DAMs were screened with a threshold value of FC ≥1.5 (or ≤0.7), VIP >1, and *p*-value ≤0.05 (**Supplementary Table S1**). The results of a hierarchical clustering analysis of these DAMs are presented as heatmaps in **Figures 2C, D**, wherein the shorter the cluster branch, the higher the similarity of the samples. The number of DAMs that were found to be significantly upregulated (colored red in **Figures 2C, D**) was 317, while 171 of them were found to be significantly downregulated (colored blue in **Figures 2C, D**). These DAMs were subsequently divided into different subclasses (**Supplementary Table S2**). The metabolites in the TF1-grown samples that were significantly upregulated mainly corresponded to amino acids, peptides and analogs, benzoic acids and derivatives, purine ribonucleotides, pyridinecarboxylic acids and derivatives, and indolyl carboxylic acids and derivatives. Carbohydrates and carbohydrate conjugates, fatty acid esters, fatty amides, and triterpenoids were mostly downregulated in the TF1-grown samples compared with those grown on TY3. In the subclass of “carbohydrates and carbohydrate conjugates,” the number of upregulated DAMs in TY3-grown fungi is twice the number of downregulated DAMs in TF1, which may lead to the polysaccharide content being higher in the TY3-grown fungi. The results of the proteomics analysis were then used to try to identify the enzymes responsible for these metabolic changes.

### 3.3 Overview of the proteomics results

**Figure 3:**
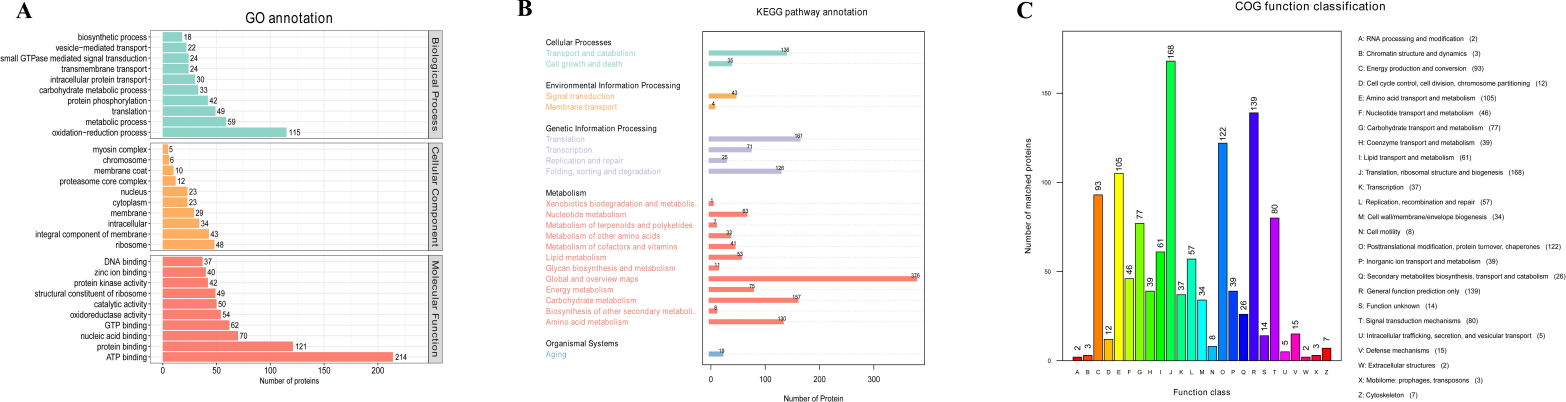
Results of the GO, KEGG, and COG analyses. **(A)** GO analysis, **(B)** KEGG analysis, and **(C)** COG analysis.

A TMT-based proteomic approach was implemented to compare T. fuciformis cultivated on the mixed substrate TF1 and the *Cyclobalanopsis* substrate TY3. A total of 6,736 peptides were identified, which were assembled into 1,685 proteins (**Supplementary Table S3**). GO, COG, and KEGG analyses were subsequently performed to acquire more information about the function of these proteins, giving the results shown in **Figure 3**.

The GO results (**Figure 3A**) demonstrate the function and enrichment levels of 1,167 proteins associated with various biological processes and cellular features. The oxidation–reduction process was found to be the most enriched (115) among the biological processes. These, presumably, generate energy for other processes, such as metabolic processes (59), translation (49), protein phosphorylation (42), and carbohydrate metabolic processes (33). This outcome is consistent with the finding that the number of proteins associated with ATP binding is the most enriched (214) category in the molecular function group, as well as protein binding (121), nucleic acid binding (70), GTP binding (62), oxidoreductase activity (54), and catalytic activity (50). In the category of cellular components, the main differences involve proteins associated with membrane structure, e.g., integral components of the membrane (43), membrane (29), cytoplasm (23), and membrane coat (10).

The KEGG pathway results (**Figure 3B**) show that the 1,685 proteins enrich 21 pathways. The metabolic pathways were the most enriched, especially pathways related to the metabolism of carbohydrates (157) and amino acids (130). Other, less significant, changes were related to genetic information processing, cellular processes, environmental information processing, and organismal systems.

To collect more information regarding the functions of each protein, a COG analysis was implemented (**Figure 3C**). In this case, 1,092 proteins were classified into 25 categories. The proteins most enriched were related to translation, ribosomal structure, and biogenesis (168); post-translational modification, protein turnover, and chaperones (122); and energy production and conversion (93), reflecting a status of hyperactive expression. The following functions were also enriched: amino acid transport and metabolism (105) and carbohydrate transport and metabolism (77). This coincides with the aforementioned changes in metabolic function.

Threshold values of FC ≥1.2 (or ≤0.8) and p ≤0.05 were set to determine the DEPs in the T. fuciformis grown on TF1 and TY3. As a result, 668 DEPs were identified, 345 of which were upregulated, and the remainder (323) were downregulated (**Figure 2E**). To elucidate the main biochemical metabolic pathways and signal transduction pathways the DEPs were associated with, the DEPs were categorized into pathways they were involved with: 66 significantly enriched pathways were thus found (**Supplementary Table S4**). The DEPs mostly enriched were related to the metabolism of amino acids, carbohydrates, lipids, cofactors, and vitamins.

With regard to carbohydrate metabolism, the DEPs are implicated in diverse pathways, including the pentose phosphate pathway, ascorbate and aldarate metabolism, starch and sucrose metabolism, pyruvate metabolism, inositol phosphate metabolism, propanoate metabolism, and fructose and mannose metabolism (also encompassing the biosynthesis of various N-glycans). With respect to amino acid metabolism, the prominent DEPs are primarily engaged in the biosynthesis and/or metabolic processes of lysine, arginine, tryptophan, tyrosine, histidine, proline, alanine, aspartate, glutamate, and phenylalanine. In terms of lipid metabolism, the DEPs are associated with the synthesis and degradation of fatty acids and ketone bodies, as well as the metabolism of arachidonic acid, ether lipids, steroids, and glycerolipids. Additionally, other notable DEPs play crucial roles in maintaining regular physiological activities (e.g., replication and repair, translation, folding, sorting and degradation, cell growth and death, transport and catabolism, etc.).

### 3.4 CAZyme annotation of the DEPs

SThe CAZyme database offers information about enzymes that degrade, modify, or create glycosidic bonds (and also conjugation to nucleic acid, lipids, polyphenols, proteins, and other compounds) (Ma et al., 2021). In our study, 161 enzymes in proteomes were annotated using the CAZyme database (**Supplementary Table S5**), and 67 of them were DEPs (**Table 1**). These DEPs were related to carbohydrate synthesis [via glycosyltransferases (GTs)], carbohydrate degradation [glycoside hydrolases (GHs), carbohydrate esterases (CEs), polysaccharide lyases (PLs), and auxiliary activities (AAs)], and carbohydrate recognition [carbohydrate-binding module (CBM)] (André et al., 2014).

**Table 1:** Annotations of DEPs derived using the CAZyme database.

Upregulated (in TF1- vs. TY3-grown fungi)			Downregulated (in TF1- vs. TY3-grown fungi)		
Protein ID	CAZyme family	Possible function	Protein ID	CAZyme family	Possible function
Glycoside hydrolases (GHs)					
J9VRQ0	GH5_22	Cellulase, hemicellulase (endo-β-1,4-glucanase)	F5H989	GH71	α-1,3-Glucanase
A0A226BGM6	GH5_12	Cellulase (steryl β-glucosidase, β-glucosidase, β-glucosylceramidase)	Q5KGL9	GH5_9	Cellulase (β-glucosidase, exo-β-1,3-glucanase, endo-β-1,6-glucanase)
A0A226BL33	GH47	Mannosidase	A0A225YW44	GH5_12	Cellulase (steryl β-glucosidase, β-glucosidase, β-glucosylceramidase)
J9VKI8	GH43_30	Pectinases (β-D-galactofuranosidase)	A0A225XMW5	GH18	Chitinase
A0A225XP04	GH37	Trehalase	A0A226BQT7	GH18	Chitinase
J9VZB3	GH3	Cellulase (β-glucosides)	Q5KE51	GH18	Chitinase
J9VQB5	GH18	Glyco_hydro_18	J9VPG5	GH13_5	α-Amylase
Q5KH58	GH152	Glucanase	Q5KLA7	GH13_31	α-Amylase
J9VLL9	GH15	Glycosyl hydrolase	Q5KA48	GH13_25+GH133	Glycogen debranching system (amylo-α-1,6-glucosidase)
Q55KB2	GH13_1	α-Amylase	Q5KCX7	GH12	Hemicellulase (xyloglucan hydrolase)
Q5KNB3	CBM48+GH13_8	1,4-α-Glucan branching enzyme	F5HHP5	CBM13+GH5-54	Cellulase
			Q5KE51	GH18	Chitinase
Glycosyltransferases (GTs)					
F5HFB2	GT30	α-3-deoxy-D-manno-octulosonic-acid (KDO) transferase	J9VI11	GT30	α-3-deoxy-D-manno-octulosonic-acid (KDO) transferase
B9U0G3	GT3	Glycogen (starch) synthase	Q5K7W2	GT95	Purine-nucleoside phosphorylase
Q8J2S9	GT48	1,3-β-Glucan synthase	A0A226BG17	GT22	α-1,2-Mannosyltransferase
F5HH45	GT35	Glycogen or starch phosphorylase	Q5KA27	GT4	sucrose synthase
A0A226BQ63	GT20	α,α-Trehalose-phosphate synthase	F5HCK5	GT20	α,α-Trehalose-phosphate synthase (UDP-forming)
F5HDU0	GT3	Glycogen (starch) synthase	F5HB36	GT2	Cellulose synthase
A0A225ZJW3	GT1	UDP-glucuronosyltransferase	F5HAT2	GT69	α-1,3-Mannosyltransferase
A0A225Z331	GT31	β-1,3-GalNAc transferase	A0A226BNX7	GT71	α-Mannosyltransferase
A0A225Y112	GT66	Dolichyl-diphosphooligosaccharide—protein glycotransferase	A0A226A056	GT69	α-1,3-Mannosyltransferase
Q5KKP8	GT0		Q5WQW6	GT90	(Mannosyl) glucuronoxylomannan/galactoxylomannan β-1,2-xylosyltransferase
			J9VI09	GT47	Xyloglucan β-galactosyltransferase
			Q5KP26	GT87	α-1,2-Mannosyltransferase
			A0A225Y8X3	GT22	α-1,2-Mannosyltransferase
			J9VIY8	GT1	Rhamnosyltransferase
			Q5KBD0	GT2	Cellulose synthase
			Q5KFM3	GT4	Sucrose synthase
			A0A225YIU0	GT1	Rhamnosyltransferase
			Q5K7J7	GT0	Starch-binding domains (SBD)
Polysaccharide lyases (PLs)					
A0A225XUM6	PL38	Glucuronan lyase			
Carbohydrate esterases (CEs)					
A0A225ZTU9	CE8	Pectin methylesterase			
Auxiliary activities (AAs)					
Q5KGP3	AA9(GH61)	Lytic cellulose monooxygenase (cellulase)	A0A226BGZ3	AA2	Lignin peroxidase
A0A225YY13	AA2	Lignin peroxidase			
J9VW95	AA7(132-337)/AA4_e1	Glucooligosaccharide oxidase			
Carbohydrate-binding modules (CBMs)					
A0A225ZYY5	CBM12	Cellulose-binding domain family XII	A0A225Y977	CBM21	Glycogen-binding function, appended to GH13 modules
Q5KET5	CBM48	Glycogen-binding function, appended to GH13 modules	A0A225YK20	CBM48	Cleaving either chitin or peptidoglycan
A0A225ZLK2	CBM48	Also found in the beta subunit (glycogen-binding) of AMP-activated protein kinases (AMPK)	Q5KCV6	CBM50	Cleaving either chitin or peptidoglycan
Q5KNB3	CBM48+GH13_8	Glycogen-binding function, appended to GH13 modules	Q5K8Q3	CBM50	Bind galactose residues, xylan, corresponding module of GalNAc transferase
			F5HHP5	CBM13+GH5	Cellulose-binding domain family XIII
			F5H989	CBM13+GH5	cellulase

More specifically, 10 enzymes related to carbohydrate synthesis (GTs) were upregulated in TF1-grown specimens compared with TY3-grown specimens, and 18 of them were downregulated. As for carbohydrate degradation, 16 enzymes were upregulated, while 13 were downregulated. Four enzymes related to carbohydrate recognition were found to be increased in TF1-grown fungi, and six were decreased in TY3-grown fungi. The TY3 Cyclobalanopsis substrate thus appeared to have a greater ability to promote carbohydrate synthesis. As a result, the fruit bodies of TY3-grown *T. fuciformis* and its soup both have higher polysaccharide contents (**Figures 1B, C**).

**Figure 4:**
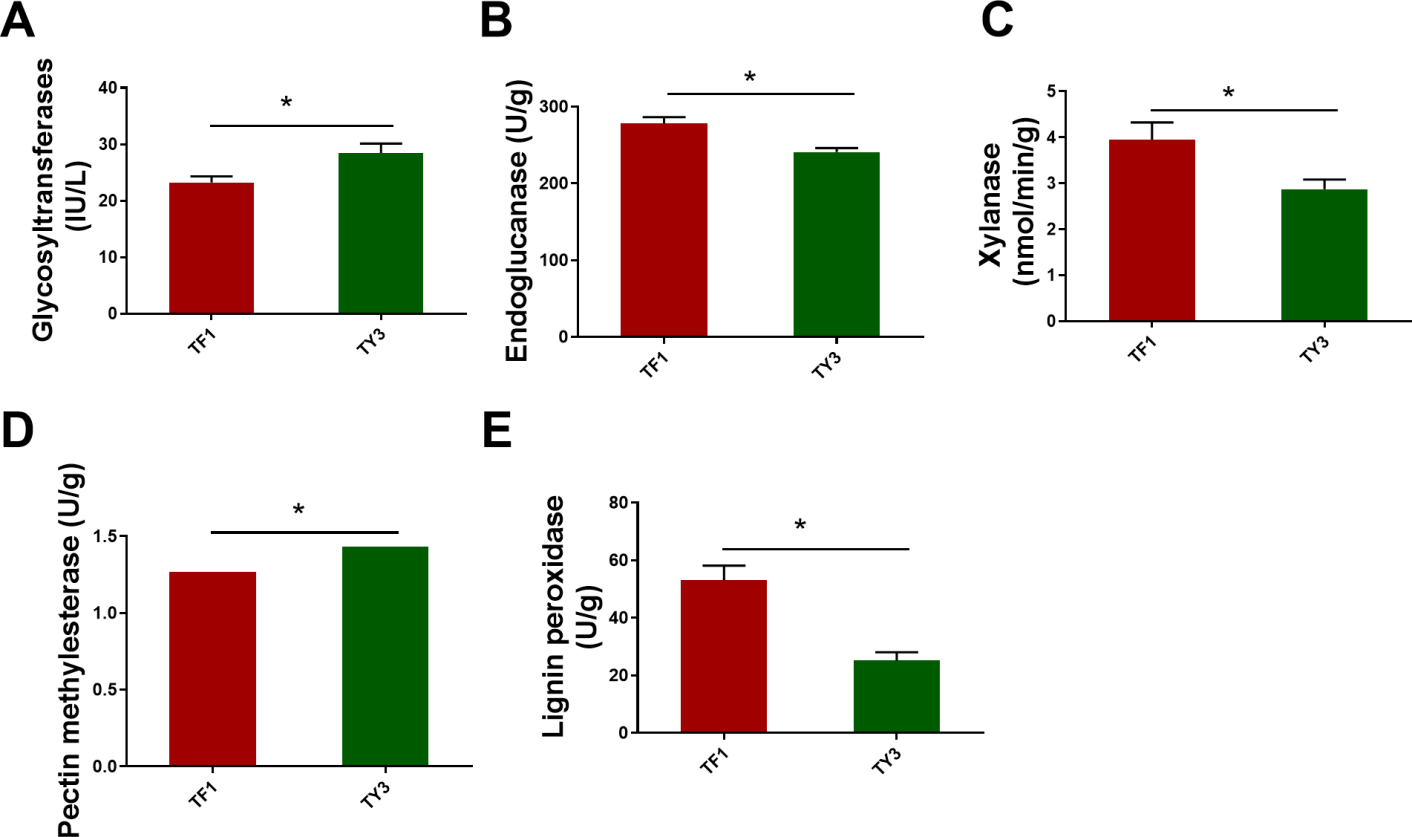
Enzyme activities related to cellulose, hemicellulose, pectin, and lignin metabolism. **(A)** Glycosyltransferases. **(B)** Cellulase (endoglucanase). **(C)** Xylanase. **(D)** Pectin methylesterase. **(E)** Lignin peroxidase. The numerical values correspond to mean ± SD (three replications), and significant differences (i.e., p < 0.05) between the TF1 and TY3 results are marked using asterisks.

The polysaccharides are mainly synthesized by GTs. The polysaccharides in fungal cells are mainly cellulose, hemicellulose, pectin, and lignin. GT2 occupies a dominant position in the synthesis of cellulose (Wei et al., 2015). The annotated enzymes of cellulose synthesis (F5HB36, Q5KBD0) belonging to GT2 were both downregulated in TF1-grown *T. fuciformis* compared with the TY3-grown specimens (**Table 1**). The synthesis of hemicellulose is more complicated than that of cellulose because of its more complex structure. Therefore, not only GT2 but also GT8, GT34, GT37, GT43, GT47, and GT61 were involved in hemicellulose synthesis (Dawn et al., 2012; Zabotina et al., 2012; Pauly et al., 2013). Xyloglucan β-galactosyltransferase (GT47, J9VI09), which adds a galactose side chain to the xyloglucan skeleton of hemicellulose and also participates in the side chain synthesis of xylose polygalacturonic acid, was found to be downregulated in **Table 1** (in TF1-grown fungi compared with TY3) (Zabotina et al., 2012; Wei et al., 2015). TY3-grown specimens having a higher content of polysaccharides may therefore be related to more GTs being upregulated in the TY3-grown *T. fuciformis* (compared to that with TF1 specimens). ELISA tests of the GTs also verified that TY3 species have higher GT activity than TF1 species (**Figure 4A**).

**Figure 5:**
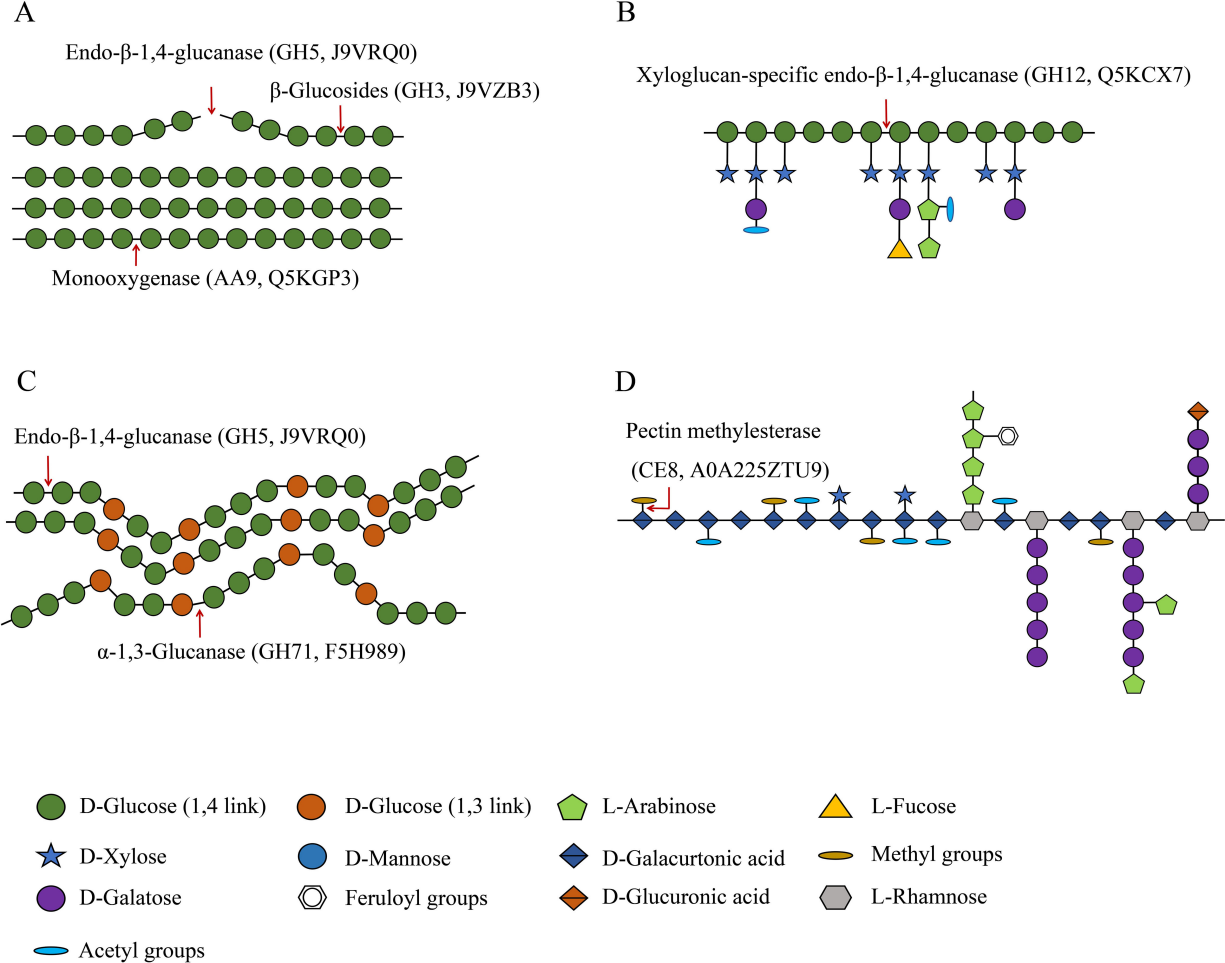
Schematic of cellulose, hemicellulose, and pectin degradation (Wei et al., 2015). **(A)** Cellulases. **(B)** Xyloglucan hydrolase. **(C)** Degradation enzymes of β-(1,3;1,4)-D-glucan. **(D)** Pectin methylesterase.

Fungi possess a variety of enzymatic systems that play different roles in degrading the cell walls of different plants (Sarkar et al., 2009). This includes *T. fuciformis* when it vegetates on Cyclobalanopsis bed log or a mixture consisting of wheat bran and cottonseed hull. The cell walls of plants mainly comprise cellulose, hemicellulose, and pectin. Cellulose is composed of unbranched glycan chains created by the formation of β-1,4 glycosidic bonds. The CAZyme annotation results indicate that endo-β-1,4-glucanase (GH5, J9VRQ0) hydrolyzes glycosidic bonds in cellulose chains from the inside. Then, β-glucosidase (GH3, J9VZB3) can degrade the glucan chains thus formed. The auxiliary activity enzyme monooxygenase (AA9, Q5KGP3) also contributed to the degradation of lytic cellulose (**Figure 5A**) (Sweeney and Xu, 2012). The annotated endoglucanase (GH5, J9VRQ0), β-glucosidase (GH3, J9VZB3), and monooxygenase (AA9, Q5KGP3) were all upregulated in TF1-grown fungi compared to TY3-grown fungi (**Table 1**). The test results obtained for the cellulase activity (endo-β-1,4-glucanase, EC 3.2.1.4) also verified that it was elevated in TF1-grown fungi compared to TY3-grown fungi (**Figure 4B**). Hemicellulose has a different skeletal and more complex side chain structure than cellulose. Its main chain structure takes many forms, including xylan, galactomannan, xyloglucan, β-(1,3;1,4)-D-glucan, and other types. The annotated xyloglucan-specific endo-β-1,4-glucanase (GH12, Q5KCX7) can degrade the xyloglucan skeleton (Master et al., 2008) (**Figure 5B**). The hemicellulose skeleton of β-(1,3;1,4)-D-glucan, like cellulose, is composed of β-D-glucose; however, due to the differences between the bond types formed during their synthesis, except endo-β-1,4-glucanase (GH5, J9VRQ0), α-1,3-glucanase (GH71, F5H989) is also needed in the degradation of β-(1,3;1,4)-D-glucan (Mc et al., 2003; Boyce and Walsh, 2007) (**Figure 5C**). The enzymes required for the degradation of the main hemicellulose chain—xyloglucan-specific endo-β-1,4-glucanase (GH12, Q5KCX7) and α-1,3-glucanase (GH71, F5H989)—are both downregulated in TF1-grown fungi compared to those in TY3-grown fungi (**Table 1**). There are five annotated degradation enzymes of hemicellulose belonging to GH5, and two of them (J9VRQ0, A0A226BGM6) were upregulated, while three of them (Q5KGL9, A0A225YW44, F5HHP5) were downregulated (**Table 1**). The hemicellulose in nature is mainly xylan, and the activity of xylanase (EC 3.2.1.8) was detected to be upregulated in TF1-grown fungi compared to TY3-grown fungi (**Figure 4C**).

Pectins possess the most complex polysaccharide structures in plant cell walls. Four main types are based on polygalacturonic acid (HG), xylose polygalacturonic acid (XG), rhamnose polygalacturonic acid I (RG-I), and rhamnose polygalacturonic acid II (RG II) (Himmel et al., 2007). More than 60% of pectin is based on HG. The calcium bridges that occur between HG (Willats et al., 2001) and araban or galactan units in RG-I side chains (Zykwinska et al., 2005) and also boron bridges between RG-II side chains (Tavares et al., 2015) are the main factors influencing the pore sizes of plant cell walls. The pore size controls the level of exposure that occurs between cellulose, hemicellulose, and the hydrolytic enzyme GH, thereby affecting the efficiency of biomass degradation (Fleischer et al., 1999; Ridley et al., 2001; Tavares et al., 2015). HG-modifying enzymes (including PMEs, pectin acetylesterases, pectate lyases, and polygalacturonases) change the pectin content or branching pattern and thereby reduce the content of HG (which limits the solubility of the major components of cell walls). Thus, they increase the solubility of the pectin, xylan, and other hemicellulose components, which improves the efficiency of the enzymatic hydrolysis process used to degrade biomass (Biswal et al., 2014; Sénéchal et al., 2014; Tomassetti et al., 2015). PME (EC 3.1.1.11) excises the methoxyl groups to form free carboxyl groups on the galacturonic acid chain and methanol (**Figure 5D**). TF1-grown T. fuciformis was found to have higher PME (CE8, A0A225ZTU9) activity than the TY3-grown fungi according to proteomics results (**Table 1**). However, based on the kit tests, the PME activity in TY3-grown fungi was found to be higher than that in TF1-grown fungi (**Figure 4D**). PMEs were encoded by a super-large family of genes in several species, and CE8 was one of them. This single protein (CE8, A0A225ZTU9) on its own cannot represent the activity of the entire PME family.

## 4 Discussion

The lignin–carbohydrate complexes (formed by lignin and carbohydrate moieties becoming crosslinked via various chemical bonds) make the walls of cells form tight network structures (Chundawat et al., 2011). The efficiency with which biomass degrades therefore depends on the content and proportion of lignin and its monomers in plants, which can have different effects (Wang et al., 2016). For example, lignin prevents the cellulose microfibril from swelling, which reduces the surface area available for cellulase to function and thus restrains the cellulase activity (Keating et al., 2006). The lignin content of the TF1 mixed substrate was found to be 52.4% of that of the TY3 Cyclobalanopsis substrate (**Figure 1A**), but the activity of lignin peroxidase was found to be increased in TF1-grown *T. fuciformis* compared with TY3-grown specimens (**Figure 4**). This inverse relationship suggests that the high lignin content in TY3 may trigger a substrate-driven adaptive response in *T. fuciformis*: the production of ligninolytic enzymes with an easily metabolizable carbon source. Such a strategy aligns with the “carbon catabolite repression” hypothesis in lignocellulose-degrading fungi, where lignocellulose-rich substrates suppress lignolytic enzyme production to conserve energy (Yoav et al., 2018; Marian et al., 2022). Therefore, the higher lignin content of TY3 means that it is more difficult to degrade enzymatically. The number of other carbohydrate degradation agents annotated in TF1 was also greater than that in TY3 (**Table 1**).

The pectin content of the TF1 substrate, on the other hand, was found to be 3.7 times that of the TY3 substrate. Pectin contains a certain amount of fermentable sugar and is more water-soluble and more readily degraded than other components of the cell wall. Therefore, using biomasses with a high content of pectin can improve the amount of bioenergy that can be extracted from the raw material (Edwards and Doran, 2012). The higher pectin content of the TF1 substrate may also explain why its biomass can be degraded more easily when it is used as a growth substrate.

The monosaccharide composition of T. fuciformis was found to depend on the growth substrate used. The monosaccharides associated with the polysaccharides found in T. fuciformis are mainly mannose (making the largest contribution), glucuronic acid, glucose, galactose, xylose, and rhamnose (Ge et al., 2020; Xu et al., 2021). The *T. fuciformis* grown using *Cyclobalanopsis* bed log as a substrate has a higher mannose content compared to those grown on the mixed substrate (Gou et al., 2017). The main chain of mannan consists of α-1,6-linked mannose units with α-1,2-linked and α-1,3-linked mannose units attached as short side branches (Herscovics and Orlean, 1993; Schoffelmeer et al., 2001). Mannosyltransferase is indispensable for the synthesis of the mannan present in cell walls. The annotated α-1,2-mannosyltransferase and α-1,3-mannosyltransferase (A0A226BG17, F5HAT2, A0A226A056, Q5KP26, and A0A225Y8X3) in *T. fuciformis* were all found to be upregulated in the TY3-grown fungi. As *T. fuciformis* specimens cultivated using TY3 had a higher mannose content (compared to specimens grown using TF1), we suppose that cultivating *T. fuciformis* on a substrate of *Cyclobalanopsis* bed log upregulates the expression of α-1,2-mannosyltransferase and α-1,3-mannosyltransferase and thus promotes the synthesis of mannan.

Cellulose synthesis (F5HB36, Q5KBD0) belonging to GT2 is downregulated in TF1-grown T. fuciformis compared with the TY3-grown specimens. Moreover, the cellulase annotated in TF1-grown T. fuciformis was found to be upregulated, and the activity of cellulase (endo-β-1,4-glucanase, EC 3.2.1.4) was also found to be elevated in TF1-grown specimens compared to TY3-grown specimens (**Figure 4B**). The downregulation of cellulases and the upregulation of cellulose synthases (GT2 family) in TY3-grown fungi suggest a substrate-mediated shift from degradation to de-novo synthesis. This aligns with the “substrate feedback regulation” observed in Agaricus bisporus, where a high lignocellulose content of the substrate results in a higher yield of A. bisporus (Wang et al., 2023).

The enzymes capable of degrading hemicellulose, xyloglucan-specific endo-β-1,4-glucanase (GH12, Q5KCX7), α-1,3-glucanase (GH71, F5H989), and endoglucanases (GH5, Q5KGL9, A0A225YW44, and F5HHP5) that were annotated were found to be downregulated in TF1-grown T. fuciformis compared to those grown on TY3. However, the glycoside hydrolases (GH5, J9VRQ0, and A0A226BGM6) were upregulated. There are many kinds of hemicellulases, including xylanase (EC 3.2.1.8), mannase, and different glycosidases that can degrade the hemicellulose main chain (as well as arabinosidases, acetylxylanesterases, glucuronidases, and other enzymes that can degrade side chains) (Scheller and Ulvskov, 2010). The hemicellulose found in nature mainly consists of xylan (Schoffelmeer et al., 2001). Although the testing results of the xylanase (EC 3.2.1.8) activity test showed increased activity in TF1-grown T. fuciformis compared to the TY3-grown variety (**Figure 4C**), it is hard to ascertain what difference arises in the total hemicellulase activity.

PME (EC: 3.1.1.11) is widely found in bacteria, fungi, and higher plants. It belongs to the carbohydrate esterase family CE8 and is encoded by a large family of genes in many species. PME family members have different expression patterns in different developmental stages, tissues, organs, and physiological states of plants (Jolie et al., 2010). The activity of PME depends on the pH of the cellular environment. Most plants and bacteria have PMEs whose isoelectric points are neutral or alkaline, and a few plants and fungi have PMEs whose isoelectric points are acidic or neutral (Sénéchal et al., 2014). Mediated by acidic PMEs in fungi, demethylated HG tends to activate pectin-degrading enzymes and depolymerize the long chains in HG; thus, acidic PMEs loosen cell walls (otherwise highly unesterified HG can be crosslinked with Ca^2+^ ions to form “egg-box” structures that reinforce the cell walls) (Pelloux et al., 2007; Sénéchal et al., 2014; Wolf et al., 2014). The activity of PME in TY3-grown fungi was found to be higher than that in TF1-grown fungi (**Figure 4D**). Therefore, in acid mode, PME makes the cell walls of *T. fuciformis* grown using TY3 looser. This made the TY3 easier to braise and dissolve, so that the dissolved colloid content of the soup derived from TY3-grown fungi was greater than that of the TF1-derived soup with the same cooking time (**SFigure 1**). This may also be the reason why the TY3-grown *T. fuciformis* produced soup was more viscous. PMEs were encoded by a super-large family of genes in several species. In this work, the expression of the PME A0A225ZTU9 (CE8 family) was found to be upregulated in TF1-grown fungi. Although this contradicts the results of the PME activity test, we note that this single CE8-family protein (A0A225ZTU9) cannot fully represent the activity of the entire PME family.

The discrepancy between proteomic data (indicating upregulation of PME A0A225ZTU9 in TF1-grown fungi) and ELISA assays (showing higher PME activity in TY3-grown fungi) likely arises from the biological complexity in PME family diversity and post-translational regulation mechanisms. PME is encoded by a large gene family with functional redundancy and substrate specificity. Proteomics identified only A0A225ZTU9 (CE8 family), but other PME isoforms may dominate the activity in TY3. The TY3 substrate may induce the expression of acidic PMEs, which are more effective in loosening the cell wall. These isoforms might not have been detected by proteomics due to low abundance or technical limitations. Post-translational modifications including phosphorylation or glycosylation play an essential role in most biological processes (Duan et al., 2023). Phosphorylation or glycosylation of PMEs can alter their activity without affecting abundance. TY3-grown fungi may exhibit post-translational activation of PMEs (e.g., phosphorylation at catalytic sites), enhancing activity despite lower proteomic detection. TF1-grown fungi might accumulate inactive PME precursors that are detected by proteomics but not functional in assays.

The dynamic interplay between CAZymes and fungal polysaccharide metabolism is induced by the lignocellulose content of the substrate. There is a large amount of CAZymes, and their number has increased steadily nowadays (Drula et al., 2021). Each of the polysaccharide synthesis or degradation pathways has its own independent regulatory systems, and there are interactions between these pathways (AlJourani et al., 2023; Bains et al., 2023; Bains et al., 2024). Target of rapamycin (TOR) signaling is the center of regulating cell growth in response to nutrient availability of organisms (Weisman, 2016). Inhibition of TOR signaling reduced the growth of *Ganoderma lucidum* and increased the content of β-1,3-glucan in the cell wall via the Slt2–MAPK pathway (Chen et al., 2019). To date, very little is known about the precise regulation mechanisms for *T. fuciformis*. In the future, we will further elucidate the regulatory mechanisms of different lignocellulose content substrates on polysaccharide synthesis in *T. fuciformis* from the perspective of CAZyme-specific enzymes and their regulatory pathways.

The observed differences in CAZyme expression and activity between *T. fuciformis* grown on TF1 and TY3 substrates could be influenced by several confounding factors related to nutrient availability or environmental stress. TY3 (lignocellulose-rich): high cellulose (41.2%), hemicellulose (28.5%), and lignin (19.8%) but low pectin (4.5%) (**Figure 1A**). Lignocellulose-rich substrates like TY3 are typically nitrogen-poor. This lignocellulose dominance may force *T. fuciformis* to prioritize polysaccharide synthesis over degradation to conserve energy. A similar situation also occurs in the cultivation of button mushrooms (*A. bisporus*). Changes in carbon and nitrogen sources in compost can significantly alter the yield and quality of button mushrooms, for instance, by changing the N content in the caps of button mushrooms (Noble et al., 2024). Lignin degradation generates reactive oxygen species (ROS), a kind of oxidative stress, which may repress lignolytic enzymes (lignin peroxidase) via redox imbalance, as observed in TY3 (**Figure 4E**), and induce antioxidant pathways (glutathione synthesis), diverting resources from polysaccharide biosynthesis (Blokhina et al., 2003). While substrate lignocellulose content is a key driver of CAZyme regulation, nutrient availability and environmental stress are critical confounding factors. These variables likely synergize to shape the observed metabolic shift toward polysaccharide biosynthesis in TY3-grown fungi. Future studies should systematically control these factors to refine the mechanistic understanding of CAZyme regulation in *T. fuciformis*.

The findings from this study provide actionable insights for enhancing *T. fuciformis* cultivation efficiency, substrate design, and strain improvement; optimizing the substrate to balance polysaccharide synthesis (via GT upregulation) and growth rate (via partial pectin degradation); providing molecular breeding targets for strain improvement, including enhancing GT activity and repressing GH/AA9 expression; and adopting rapid CAZyme activity assays for batch monitoring to conduct quality control metrics for industrial production ensuring batch consistency and high polysaccharide output.

## 5 Conclusions

The variations in the lignocellulose content of *Cyclobalanopsis* log and the mixed substrate influenced the polysaccharide content of *T. fuciformis* grown on these substrates. Notably, *T. fuciformis* grown on the mixed substrate exhibited a reduced polysaccharide content compared to those cultivated solely on *Cyclobalanopsis* log. Furthermore, LC-MS/MS analysis revealed a substantial downregulation of metabolites associated with carbohydrates and their conjugates in TF1-cultured samples. When considered alongside proteomics data indicating the highest protein enrichment in the carbohydrate metabolic pathway, these findings are complemented by notable upregulation of carbohydrate synthases in TY3-cultured species, as annotated using the CAZyme database. Additionally, the upregulation of α-1,2-mannosyltransferase and α-1,3-mannosyltransferase—enzymes that facilitate mannose synthesis—suggests a superior capacity of the TY3 substrate to stimulate carbohydrate synthesis. The elevated activity of PME observed in TY3-cultured samples implies that the cell walls of *T. fuciformis* grown on this substrate are likely to be more relaxed, facilitating easier cooking and dissolution of these mushrooms, which contributes to increasing polysaccharide-mediated viscosity. The enzymatic activity in TF1-grown fungi with respect to lignocellulose degradation (cellulase, xylanase, and lignin peroxidase) was found to be increased compared to when TY3 was used. This implies that *T. fuciformis* could degrade the carbohydrates in the TF1 substrate more efficiently. This study provides metabolomic and proteomic insights into the polysaccharide content in *T. fuciformis* grown using different substrates. As such, it should help to improve the quality of *T. fuciformis* produced industrially. It may also yield simplified protocols for polysaccharide content control in industrial testing.

## Data Availability

The original contributions presented in the study are publicly available. This data can be found here: MetaboLights, http://ftp.ebi.ac.uk/pub/databases/metabolights/studies/public/MTBLS12694 and iProX, https://www.iprox.cn/page/SSV024.html;url=1751366101678rjq7 password zMRo.
