# Supplementary figures and images for: Lignocellulose adaptation drives polysaccharide biosynthesis in Tremella fuciformis: metabolomic and proteomic insights into CAZyme regulation
Source: Front Fungal Biol. 2025 Jul 11;6:1617458. doi: 10.3389/ffunb.2025.1617458 (PMC12289600; doi:10.3389/ffunb.2025.1617458)

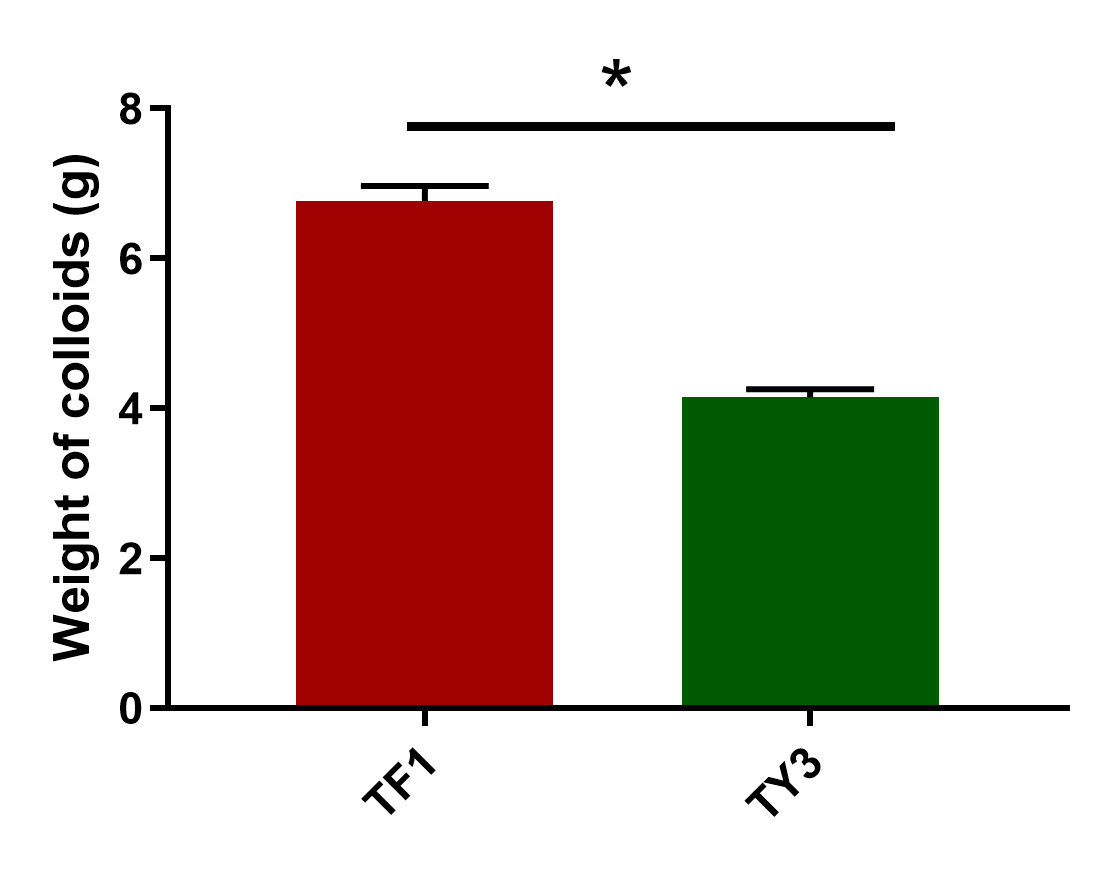

Supplement: Supplementary file 1 [file Image1.tif]
