# Supplementary material for: Lignocellulose adaptation drives polysaccharide biosynthesis in Tremella fuciformis: metabolomic and proteomic insights into CAZyme regulation
Source: Front Fungal Biol. 2025 Jul 11;6:1617458. doi: 10.3389/ffunb.2025.1617458 (PMC12289600; doi:10.3389/ffunb.2025.1617458)
Supplement: Supplementary file 4 [file Table3.docx]

**Table 1.** Sub-class and number of up- and down-regulated DAMs in each sub-class (in TF1-grown compared to TY3-grown *T. fuciformis*).

| Up-regulated DAMs | | Down-regulated DAMs | |
| --- | --- | --- | --- |
| Sub-class | Number | Sub-class | Number |
| Amino acids, peptides, and analogues | 44 | Fatty acids and conjugates | 11 |
| Fatty acids and conjugates | 10 | Carbohydrates and carbohydrate conjugates | 6 |
| Benzoic acids and derivatives | 6 | Fatty acid esters | 4 |
| Purine ribonucleotides | 6 | Amino acids, peptides, and analogues | 4 |
| Pyridinecarboxylic acids and derivatives | 4 | Fatty amides | 3 |
| Indolyl carboxylic acids and derivatives | 4 | Triterpenoids | 3 |
| Pyrimidine 2'-deoxyribonucleosides | 3 | Methoxyphenols | 3 |
| Hydroxyindoles | 3 | Bile acids, alcohols and derivatives | 3 |
| Carbohydrates and carbohydrate conjugates | 3 | Hydroxycinnamic acids and derivatives | 2 |
| Eicosanoids | 3 | Hydroxycoumarins | 2 |
| Pyrimidines and pyrimidine derivatives | 2 | Retinoids | 2 |
| Alcohols and polyols | 2 | Benzoic acids and derivatives | 2 |
| Hybrid peptides | 2 | Pyrimidine deoxyribonucleotides | 1 |
| Estrane steroids | 2 | Glycerophosphocholines | 1 |
| Hydroxysteroids | 2 | Carbonyl compounds | 1 |
| Androstane steroids | 2 | Purines and purine derivatives | 1 |
| Phenethylamines | 2 | Quinone and hydroquinone lipids | 1 |
| Quaternary ammonium salts | 2 | Medium-chain keto acids and derivatives | 1 |
| Pyrimidine ribonucleotides | 2 | Gamma-keto acids and derivatives | 1 |
| Lipoic acids and derivatives | 2 | Cyclic alcohols and derivatives | 1 |
| Fatty acid esters | 2 | Benzenediols | 1 |
| Indoles | 2 | Purine nucleotide sugars | 1 |
| Imidazoles | 1 | Furanones | 1 |
| Tryptamines and derivatives | 1 | Alloxazines and isoalloxazines | 1 |
| Lipoamides | 1 | Nicotinamide nucleotides | 1 |
| Ergostane steroids | 1 | Purine 2'-deoxyribonucleosides | 1 |
| Glycerophosphocholines | 1 | Short-chain keto acids and derivatives | 1 |
| Purines and purine derivatives | 1 | 1-hydroxy-2-unsubstituted benzenoids | 1 |
| Flavans | 1 | Eicosanoids | 1 |
| Amines | 1 | Phenylpropanes | 1 |
| Piperazines | 1 | Androstane steroids | 1 |
| Coumestans | 1 | Others | 107 |
| Anisoles | 1 |  |  |
| Steroid esters | 1 |  |  |
| Pyrrole carboxylic acids and derivatives | 1 |  |  |
| Carbonyl compounds | 1 |  |  |
| Methoxyphenols | 1 |  |  |
| Phenylacetamides | 1 |  |  |
| Monoterpenoids | 1 |  |  |
| Sesquiterpenoids | 1 |  |  |
| Non-metal phosphates | 1 |  |  |
| Lineolic acids and derivatives | 1 |  |  |
| Gamma butyrolactones | 1 |  |  |
| Linear diarylheptanoids | 1 |  |  |
| Polyols | 1 |  |  |
| Phenols and derivatives | 1 |  |  |
| Medium-chain keto acids and derivatives | 1 |  |  |
| Benzenediols | 1 |  |  |
| Quinoline carboxylic acids | 1 |  |  |
| Hydroxycinnamic acids and derivatives | 1 |  |  |
| Cyclamates | 1 |  |  |
| Pyridoxines | 1 |  |  |
| Medium-chain hydroxy acids and derivatives | 1 |  |  |
| Others | 176 |  |  |
