# Supplementary material for: Lignocellulose adaptation drives polysaccharide biosynthesis in Tremella fuciformis: metabolomic and proteomic insights into CAZyme regulation
Source: Front Fungal Biol. 2025 Jul 11;6:1617458. doi: 10.3389/ffunb.2025.1617458 (PMC12289600; doi:10.3389/ffunb.2025.1617458)
Supplement: Supplementary file 6 [file Table5.docx]

Table S4. KEGG pathways the DEPs enriched.

| Kegg_level2 | MapID | MapTitle | Pvalue | AdjustedPv | x | y | n | N | ProtID |
| --- | --- | --- | --- | --- | --- | --- | --- | --- | --- |
| Amino acid metabolism | map00310 | Lysine degradation | 0.000970158 | 0.097985989 | 13 | 16 | 337 | 878 | F5HF96 Q6RXX2 F5HB36 Q5K8N2 Q5KMP3 Q55PN9 F5HAJ5 Q5KIV9 A0A226BKJ7 F5HCT2 Q5K8N4 Q5KBZ0 Q55YZ3 |
|  | map00300 | Lysine biosynthesis | 0.004065167 | 0.199423896 | 9 | 11 | 337 | 878 | Q6RXX2 A0A225XHB0 A0A225ZHU4 A0A225Y3I9 J9VKX7 A0A226BKJ7 Q55N96 Q5KM49 A0A226BAJ2 |
|  | map00340 | Histidine metabolism | 0.016534093 | 0.417485843 | 8 | 10 | 337 | 878 | F5HF96 Q5K8N2 J9VGN9 F5HCT2 A0A226B6W8 Q5KIW5 Q55YZ3 A0A226BPU7 |
|  | map00330 | Arginine and proline metabolism | 0.04504412 | 0.758242684 | 10 | 15 | 337 | 878 | F5HF96 A0A226BCS0 J9VP99 Q5K8N2 J9W469 J9VKH1 F5HCT2 A0A225YPB4 Q55RP6 Q55YZ3 |
|  | map00380 | Tryptophan metabolism | 0.081405535 | 0.913550999 | 10 | 16 | 337 | 878 | F5HF96 F5HB36 Q5K8N2 Q55PN9 F5HAJ5 F5HCT2 A0A225YPB4 Q5KM49 Q55ZE8 Q55YZ3 |
|  | map00350 | Tyrosine metabolism | 0.093797589 | 0.938947344 | 6 | 9 | 337 | 878 | A0A226BCS0 Q5KIV9 J9VGN9 Q5K7P9 Q5KM49 Q5KBZ0 |
|  | map00250 | Alanine, aspartate and glutamate metabolism | 0.161689172 | 0.938947344 | 13 | 24 | 337 | 878 | Q5KNM2 A0A226B792 A0A225XP48 A0A226BCS0 F5H9T7 A0A226BJT0 A0A225YVJ1 J9W469 Q5KIV9 J9VI09 J9VH88 F5HE83 Q5KBZ0 |
|  | map00400 | Phenylalanine, tyrosine and tryptophan biosynthesis | 0.230106465 | 0.938947344 | 7 | 12 | 337 | 878 | A0A226BCS0 A0A226BEK7 J9VGN9 J9VY08 J9VKF7 Q5KM49 Q55MJ1 |
|  | map00360 | Phenylalanine metabolism | 0.438113739 | 1 | 4 | 7 | 337 | 878 | A0A226BCS0 J9VGN9 A0A225YPB4 Q5KM49 |
|  | map00220 | Arginine biosynthesis | 1 | 1 | 4 | 10 | 337 | 878 | A0A225XP48 A0A226BCS0 A0A225YVJ1 F5HE83 |
| Carbohydrate metabolism | map00053 | Ascorbate and aldarate metabolism | 0.074615752 | 0.913550999 | 4 | 5 | 337 | 878 | F5HF96 Q5K8N2 F5HCT2 Q55YZ3 |
|  | map00500 | Starch and sucrose metabolism | 0.245660938 | 0.938947344 | 12 | 23 | 337 | 878 | Q8J2S9 A0A225XDK9 Q5KA48 F5HCK5 F5HH45 A0A226BQ63 J9VZB3 F5HDU0 Q55KB2 Q5KGL9 J9VPG5 F5HHP5 |
|  | map00620 | Pyruvate metabolism | 0.254136168 | 0.938947344 | 15 | 30 | 337 | 878 | Q55QT6 A0A226B9X8 F5HDP7 F5HF96 Q5K8N2 Q5K758 F5HAJ5 F5HDV1 J9VW95 A0A225YXV9 Q5KIM3 F5HCT2 Q55N74 J9VIE5 Q55YZ3 |
|  | map00030 | Pentose phosphate pathway | 0.435866497 | 1 | 9 | 18 | 337 | 878 | F5HFB2 J9VI11 A0A225ZZB5 A0A225XDK9 Q5K952 Q55Z44 A0A225YUF6 F5HE79 J9VZ13 |
|  | map00562 | Inositol phosphate metabolism | 0.532604478 | 1 | 7 | 14 | 337 | 878 | Q55RN1 A0A226B8L3 Q55WR9 F5HBB1 F5HAD2 F5H966 Q55WA1 |
|  | map00640 | Propanoate metabolism | 0.944163154 | 1 | 6 | 14 | 337 | 878 | Q55QT6 A0A226B9X8 A0A226BG17 F5HAJ5 F5H966 J9VLA1 |
|  | map00051 | Fructose and mannose metabolism | 1 | 1 | 7 | 18 | 337 | 878 | J9VZD5 A0A225ZZB5 A0A225XLJ7 A0A226BM86 Q55RN1 Q55PJ3 J9VZ13 |
|  | map00650 | Butanoate metabolism | 1 | 1 | 5 | 13 | 337 | 878 | F5H9T7 F5HAJ5 Q5KIV9 A0A225ZKX1 Q5KBZ0 |
| Lipid metabolism | map00590 | Arachidonic acid metabolism | 0.1470534 | 0.938947344 | 2 | 2 | 337 | 878 | Q5K7D6 A0A225YIU0 |
|  | map00071 | Fatty acid degradation | 0.350717438 | 1 | 8 | 15 | 337 | 878 | F5HF96 Q5K8N2 Q55PN9 F5HAJ5 A0A226BMX1 Q5K7P9 F5HCT2 Q55YZ3 |
|  | map00061 | Fatty acid biosynthesis | 0.438113739 | 1 | 4 | 7 | 337 | 878 | Q55QT6 A0A226B9X8 A0A225YJL6 A0A226BMX1 |
|  | map00565 | Ether lipid metabolism | 0.562131236 | 1 | 2 | 3 | 337 | 878 | Q5KHM9 J9VNL6 |
|  | map00072 | Synthesis and degradation of ketone bodies | 0.640363789 | 1 | 2 | 4 | 337 | 878 | F5HAJ5 A0A225ZKX1 |
|  | map00100 | Steroid biosynthesis | 0.68048551 | 1 | 3 | 6 | 337 | 878 | F5HGG5 A0A1I7P313 F5HI94 |
|  | map00561 | Glycerolipid metabolism | 0.775499457 | 1 | 5 | 12 | 337 | 878 | F5HF96 Q5K8N2 Q55VH9 F5HCT2 Q55YZ3 |
| Metabolism of cofactors and vitamins | map00790 | Folate biosynthesis | 0.056236174 | 0.811407661 | 3 | 3 | 337 | 878 | Q5KK71 Q5KLK7 Q55WT1 |
|  | map00760 | Nicotinate and nicotinamide metabolism | 0.210952208 | 0.938947344 | 4 | 6 | 337 | 878 | Q5KIV9 A0A225ZK23 Q55N64 Q5KBZ0 |
|  | map00770 | Pantothenate and CoA biosynthesis | 0.491437558 | 1 | 4 | 8 | 337 | 878 | A0A226BBY1 J9VWV5 Q5KJZ6 Q55S63 |
|  | map00130 | Ubiquinone and other terpenoid-quinone biosynthesis | 0.562131236 | 1 | 2 | 3 | 337 | 878 | A0A226BQ97 Q5KM49 |
|  | map00730 | Thiamine metabolism | 0.562131236 | 1 | 2 | 3 | 337 | 878 | Q5KHR6 J9VW12 |
|  | map00750 | Vitamin B6 metabolism | 0.640363789 | 1 | 2 | 4 | 337 | 878 | F5HAF4 Q5KQ01 |
|  | map00860 | Porphyrin and chlorophyll metabolism | 0.68048551 | 1 | 3 | 6 | 337 | 878 | A0A226A223 A0A225Y8X3 Q55SU1 |
| Global and overview maps | map01100 | Metabolic pathways | 0.193193003 | 0.938947344 | 151 | 368 | 337 | 878 | F5HFB2 J9VI11 Q5KNM2 A0A226BIE9 Q55QT6 A0A226B9X8 A0A225Y898 A0A226BGU0 A0A226BBY1 E3P6S3 Q55K50 J9VZD5 Q5KLA7 F5HDP7 A0A226BG17 A0A225ZZB5 A0A225YJL6 Q5K9Y3 A0A225XLJ7 A0A226B792 A0A225XDK9 Q5KA48 F5HF96 F5HCK5 Q55XC1 Q6RXX2 F5HH45 A0A226BQ63 J9VZB3 A0A225XP48 Q55RN1 A0A225XHB0 A0A226BCS0 F5HIM9 J9VP99 A0A225ZHU4 F5HDU0 F5HB36 Q55NZ3 Q5K8N2 Q5KHR6 Q5KMP3 Q5K758 Q55VH9 F5H9T7 Q55PN9 A0A226BJT0 Q5K8G6 A0A226BEK7 A0A225YVJ1 J9VWV5 F5HAJ5 A0A226BIF8 A0A226BQ97 A0A225Y3I9 F5HDV1 J9W469 J9VKX7 Q5KLA1 F5HGG5 Q55PJ3 F5HAF4 A0A226BBC4 Q5KIV9 A0A226A223 A0A225Z511 Q55VX3 Q5KPD2 A0A225ZKX1 A0A226B8L3 Q5K952 J9VI09 A0A225Y230 A0A225ZSS5 A0A226BKJ7 Q55N96 A0A226BMX1 Q55WR9 A0A225ZK23 J9VGN9 J9VY08 J9VKH1 J9VH39 J9VKF7 Q5KIM3 A0A226BJR7 Q5K7P9 Q5KJZ6 A0A226BJG2 A0A225Y8X3 Q5KHM9 A0A226B964 F5HBB1 Q55I93 Q55N64 F5HCT2 A0A225Y112 A0A226B6W8 J9VWC2 Q5KK71 Q55Z44 J9VST7 A0A225XU63 A0A225YUF6 F5HE79 F5HAD2 J9VIY8 A0A225ZZM3 A0A1I7P313 Q5KM49 F5H966 Q5KLK7 A0A225Y7F6 Q5K8N4 Q5KH62 F5HE83 Q55MJ1 J9VRV0 J9VW12 Q55HT8 Q5KGJ6 A0A226BAJ2 Q55RP6 Q5KQ01 Q55P07 Q55KB2 Q5KE51 J9VT93 Q55SU1 A0A226B778 J9VPG5 J9VU26 J9VNL6 A0A225XZA4 J9VIW7 Q5KBZ0 Q5KIW5 J9VLA1 Q55YZ3 A0A226BPU7 A0A225YIU0 Q55S63 A0A225ZZF4 J9VHY1 J9VZ13 Q55WA1 F5HI94 F5HC91 F5HHP5 A0A225XDL1 Q5KGP7 |
| Global and overview maps | map01110 | Biosynthesis of secondary metabolites | 0.354477167 | 1 | 73 | 175 | 337 | 878 | F5HFB2 J9VI11 Q55QT6 A0A226B9X8 A0A226BBY1 E3P6S3 J9VZD5 Q5KLA7 F5HDP7 A0A226BG17 A0A225ZZB5 Q5K9Y3 A0A225XLJ7 A0A226B792 A0A225XDK9 Q5KA48 F5HF96 Q6RXX2 F5HH45 J9VZB3 Q55RN1 A0A226BCS0 F5HIM9 J9VP99 F5HB36 Q5K8N2 Q5KMP3 Q55VH9 F5H9T7 A0A226BEK7 A0A225YVJ1 J9VWV5 F5HAJ5 A0A226BQ97 A0A225Y3I9 F5HDV1 F5HGG5 Q55PJ3 A0A226A223 Q55VX3 A0A225ZKX1 Q5K952 A0A226BKJ7 Q55N96 J9VGN9 J9VY08 J9VKF7 Q5KIM3 Q5K7P9 A0A225Y8X3 Q5KHM9 F5HCT2 A0A226B6W8 Q55Z44 J9VST7 F5HB77 A0A225YUF6 F5HE79 A0A1I7P313 Q5KM49 Q55MJ1 A0A226BAJ2 Q55RP6 Q55ZE8 Q55SU1 A0A226B778 J9VU26 Q5KIW5 Q55YZ3 A0A226BPU7 A0A225ZZF4 J9VZ13 A0A225XDL1 |
|  | map01230 | Biosynthesis of amino acids | 0.628938539 | 1 | 34 | 82 | 337 | 878 | A0A226BBY1 Q5KLA7 A0A225ZZB5 Q5K9Y3 Q6RXX2 Q55RN1 A0A225XHB0 A0A226BCS0 A0A225ZHU4 A0A226BEK7 A0A225YVJ1 J9VWV5 A0A225Y3I9 F5HDV1 J9VKX7 Q5K952 A0A226BKJ7 Q55N96 J9VGN9 J9VY08 J9VKF7 A0A226B6W8 Q55Z44 A0A225YUF6 F5HE79 Q5KM49 F5HE83 Q55MJ1 A0A226BAJ2 A0A226B778 J9VU26 Q5KIW5 A0A226BPU7 J9VZ13 |
|  | map01130 | Biosynthesis of antibiotics | 0.817845514 | 1 | 57 | 144 | 337 | 878 | F5HFB2 J9VI11 A0A226BBY1 Q5KLA7 A0A226BG17 A0A225ZZB5 Q5K9Y3 A0A225XDK9 F5HF96 Q6RXX2 Q55RN1 A0A225XHB0 A0A226BCS0 F5HIM9 J9VP99 A0A225ZHU4 F5HB36 Q5K8N2 Q5KMP3 Q55PN9 A0A226BEK7 A0A225YVJ1 J9VWV5 F5HAJ5 A0A225Y3I9 J9VKX7 F5HGG5 Q55VX3 Q5KPD2 A0A225ZKX1 Q5K952 A0A225Y230 A0A226BKJ7 Q55N96 J9VGN9 J9VY08 J9VKF7 Q5KIM3 Q5K7P9 F5HCT2 Q55Z44 J9VST7 A0A225YUF6 F5HE79 J9VIY8 A0A1I7P313 Q5KM49 Q5KH62 Q55MJ1 A0A226BAJ2 Q55RP6 Q55ZE8 A0A226B778 Q55YZ3 J9VZ13 F5HI94 A0A225XDL1 |
|  | map01212 | Fatty acid metabolism | 0.944163154 | 1 | 6 | 14 | 337 | 878 | Q55QT6 A0A226B9X8 A0A225YJL6 F5HAJ5 A0A226BMX1 A0A226BJA4 |
|  | map01210 | 2-Oxocarboxylic acid metabolism | 1 | 1 | 9 | 23 | 337 | 878 | A0A226BBY1 A0A225XHB0 A0A226BCS0 A0A225ZHU4 J9VWV5 F5HDV1 J9VKX7 Q55N96 Q5KM49 |
| Metabolism of other amino acids | map00410 | beta-Alanine metabolism | 0.230106465 | 0.938947344 | 7 | 12 | 337 | 878 | F5HF96 Q5K8N2 F5H9T7 F5HCT2 F5H966 J9VLA1 Q55YZ3 |
|  | map00430 | Taurine and hypotaurine metabolism | 0.562131236 | 1 | 2 | 3 | 337 | 878 | F5H9T7 A0A226B964 |
|  | map00460 | Cyanoamino acid metabolism | 0.562131236 | 1 | 2 | 3 | 337 | 878 | J9VZB3 A0A226B964 |
|  | map00480 | Glutathione metabolism | 0.775499457 | 1 | 5 | 12 | 337 | 878 | F5HFB2 J9VI11 Q5K7D6 A0A226B964 Q55HT8 |
|  | map00450 | Selenocompound metabolism | 1 | 1 | 2 | 5 | 337 | 878 | A0A225Y230 Q5KFM3 |
| Replication and repair | map03410 | Base excision repair | 0.160546244 | 0.938947344 | 3 | 4 | 337 | 878 | Q5KB11 A0A225Y7F6 J9VHY1 |
|  | map03420 | Nucleotide excision repair | 0.238783832 | 0.938947344 | 8 | 14 | 337 | 878 | Q5KB11 Q5KCE8 A0A226BFV9 A0A226BRV1 A0A225Y7F6 A0A226B6I2 J9VL39 J9VHY1 |
|  | map03440 | Homologous recombination | 1 | 1 | 2 | 5 | 337 | 878 | A0A226B7F5 J9VMD2 |
|  | map03450 | Non-homologous end-joining | 1 | 1 | 1 | 2 | 337 | 878 | A0A226B7F5 |
| Translation | map03013 | RNA transport | 0.149219185 | 0.938947344 | 18 | 35 | 337 | 878 | A0A226BCY4 Q2Q4I8 Q5KGN7 Q5KE57 A0A225Y1E9 A0A226BG02 J9VRB1 Q5KK29 F5HHN6 A0A225YY08 A0A225XLL4 F5H943 A0A225Y3J8 A0A226BFW9 Q8J0Z7 Q5KBK7 Q5KIY7 Q55QL8 |
|  | map03008 | Ribosome biogenesis in eukaryotes | 0.467555745 | 1 | 11 | 23 | 337 | 878 | Q5KGN7 Q5KE57 J9VKI8 J9VQB5 J9W3D8 F5HBV2 A0A226BB20 J9VM18 A0A226BEJ5 A0A226BC96 A0A226BET2 |
|  | map03015 | mRNA surveillance pathway | 0.980231344 | 1 | 9 | 22 | 337 | 878 | J9VMB6 A0A225X8Q1 Q5KQ04 A0A226BPE9 A0A225ZV53 Q5KPS3 A0A226B985 A0A225XYS7 Q55QL8 |
|  | map00970 | Aminoacyl-tRNA biosynthesis | 1 | 1 | 9 | 23 | 337 | 878 | F5HE46 J9VVR3 F5HAB3 A0A225Z3I0 F5HFQ4 A0A225Y8X3 A0A225ZYT8 Q5K6Z8 J9VYL9 |
| Folding, sorting and degradation | map04120 | Ubiquitin mediated proteolysis | 0.04236304 | 0.758242684 | 14 | 23 | 337 | 878 | Q5KC94 A0A225ZKW2 Q55QZ6 A0A226BAC2 A0A225XZ58 Q55QT0 A0A226B7W4 J9VMQ7 Q5K8W1 Q5KIA2 A0A226BHB1 Q5K786 Q5KBK7 J9VL39 |
|  | map04122 | Sulfur relay system | 0.383826879 | 1 | 1 | 1 | 337 | 878 | Q5KHR6 |
|  | map04141 | Protein processing in endoplasmic reticulum | 0.90184997 | 1 | 17 | 42 | 337 | 878 | A0A226B7D8 Q55QZ6 Q55QT0 A0A226B7W4 F5HGY2 J9VW50 F5HFL2 Q5K710 A0A225Y112 Q5K8W1 A0A226BRV1 A0A225XU63 J9W1U0 A0A225Y3J8 Q5KEX0 J9VL39 Q55Y28 |
| Biosynthesis of other secondary metabolites | map00254 | Aflatoxin biosynthesis | 0.1470534 | 0.938947344 | 2 | 2 | 337 | 878 | Q55QT6 A0A226B9X8 |
|  | map00261 | Monobactam biosynthesis | 0.562131236 | 1 | 2 | 3 | 337 | 878 | A0A225Y230 Q55N96 |
| Cell growth and death | map04113 | Meiosis - yeast | 0.841735764 | 1 | 9 | 21 | 337 | 878 | A0A225XMW5 A0A225X8Q1 A0A226A060 A0A225YZC7 Q5KM80 Q5K7N9 A0A226BHB1 Q8J0I6 A0A225XF56 |
|  | map04111 | Cell cycle - yeast | 1 | 1 | 10 | 25 | 337 | 878 | A0A225YL12 A0A225XMW5 A0A226B7W4 A0A225YZC7 Q5KM80 Q5KK29 A0A226BJH9 A0A226BHB1 J9VL39 Q5KDF5 |
| Energy metabolism | map00920 | Sulfur metabolism | 0.491437558 | 1 | 4 | 8 | 337 | 878 | Q5KPD2 A0A225Y230 Q5KGJ6 A0A226B778 |
|  | map00910 | Nitrogen metabolism | 1 | 1 | 3 | 7 | 337 | 878 | A0A225XP48 A0A226BIP0 F5HE83 |
| Glycan biosynthesis and metabolism | map00513 | Various types of N-glycan biosynthesis | 0.562131236 | 1 | 2 | 3 | 337 | 878 | A0A225Y112 A0A225XU63 |
|  | map00510 | N-Glycan biosynthesis | 1 | 1 | 2 | 5 | 337 | 878 | A0A225Y112 A0A225XU63 |
| Signal transduction | map04070 | Phosphatidylinositol signaling system | 0.575966463 | 1 | 6 | 13 | 337 | 878 | A0A226B8L3 Q55WR9 F5HBB1 F5HAD2 J9VMG2 Q55WA1 |
|  | map04011 | MAPK signaling pathway - yeast | 1 | 1 | 12 | 31 | 337 | 878 | Q8J2S9 Q55PJ9 A0A225YL12 A0A225XMW5 Q5KC94 A0A225YZS9 A0A226BFE0 J9VVW6 Q5KK29 F5HB77 Q55ZE8 F5HB03 |
| Transport and catabolism | map04138 | Autophagy - yeast | 0.606128211 | 1 | 17 | 39 | 337 | 878 | A0A226BEZ1 J9VH59 A0A226A060 J9VM57 F5HBB1 Q5KAF3 F5HAD2 Q5KIS3 Q5KBH1 J9VPY9 A0A226BIV0 Q8J0I6 Q5KEY7 J9VWG9 J9VMG2 A0A226BJS8 A0A226B8A6 |
| Endocrine and metabolic diseases | map04933 | AGE-RAGE signaling pathway in diabetic complications | 1 | 1 | 4 | 10 | 337 | 878 | A0A226BFE0 Q5K854 Q5KAF3 Q55WA1 |

Note: Map ID: the ID of enriched KEGG pathway; Map title: the name of enriched KEGG pathway; Pvalue: Pvalue of enrich analysis. The smaller the pvalue is, the more reliable the test is and the more statistically significant it is. x: the number of DEP related to pathway; y: the number of all proteins related to pathway; n: the number of DEP annotated with KEGG; N: the number of all proteins annotated with KEGG. ProtID: the list of enriched DEPs.

(1) MapID：富集的KEGG PATHWAY的ID ； (2) MapTitle：富集的KEGG PATHWAY名称 ； (3) Pvalue：富集分析的Pvalue ； (4) AdjustedPv：校正后的Pvalue ； (5) x：与该通路相关的差异蛋白的数目 ； (6) y：与该通路相关的背景（所有）蛋白的数目 ； (7) n：kegg注释的差异蛋白数目 ； (8) N：kegg注释的背景（所有）蛋白的数目 ； (9) ProtID：富集到的蛋白list ；
